# Association among objective and subjective sleep duration, depressive symptoms and all-cause mortality: the pathways study

**DOI:** 10.1186/s12888-025-07181-9

**Published:** 2025-07-29

**Authors:** Yuan Zeng, Tanshu Liu, Rui Qiu, Qingqing Lian

**Affiliations:** Department of Acupuncture and Moxibustion, Longyan First Affiliated Hospital of Fujian Medical University, Longyan, 364000 China

**Keywords:** Objective sleep duration, Subjective sleep duration, Depression, All-cause mortality

## Abstract

**Background:**

Sleep deprivation and overload have been associated with increased risks of both depression and mortality. However, no study has quantitatively compared the effects of objective and subjective sleep duration on mortality or examined the mediating role of depressive symptoms in these associations.

**Methods:**

Utilizing data from the NHANES 2011–2014, this study employed structural equation modeling (SEM) to explore the impact of depressive symptoms, measured by Patient Health Questionnaire (PHQ-9) scores, on the relationship between both objective and subjective sleep durations and all-cause mortality.

**Results:**

The study included 7838 participants, comprising 4392 women (55.96%) with a mean age of 46.51 (0.46) years. Over a median 6.83-year follow-up, 582 deaths occurred. The restricted cubic spline curves demonstrated a J-shaped relationship between objective sleep duration and the all-cause mortality risk, and a U-shaped relationship between subjective sleep duration and the all-cause mortality risk. SEM analysis revealed that when subjective sleep duration was < 7 h/day, the indirect effect of sleep duration on all-cause mortality was − 0.013 (*P* = 0.003), and the mediation proportion of PHQ-9 scores was 40.63%. When objective sleep duration ≥ 7 h/day, the indirect effect of sleep duration on all-cause mortality was 0.003 (*P* = 0.028), and the mediation proportion of PHQ-9 scores was 2.10%.

**Conclusions:**

The study confirmed a J-shaped and a U-shaped correlation for objective and subjective sleep duration with mortality risk. Depressive symptoms significantly mediated the association between shorter subjective sleep duration and mortality. This suggests that there is a need to focus on the co-morbidity of subjective sleep deprivation and depression.

**Supplementary Information:**

The online version contains supplementary material available at 10.1186/s12888-025-07181-9.

## Introduction

Sleep is a fundamental biological requirement intimately linked to the development and progression of various diseases, as well as mortality [1, 2]. Numerous studies substantiate a U-shaped correlation between sleep duration and mortality, indicating that both sleep deprivation and sleep overload are associated with increased mortality [3–6]. In the US, fewer than two-thirds of adults meet the recommended sleep duration of 7–9 h [7–9], underscoring the public health significance of insufficient sleep.

Meanwhile, the bidirectional relationship between sleep disorders and depression has long been recognized [10], with evidence showing that the sleep architecture and duration of patients with depression are altered, and conversely, sleep disturbances can predict the onset or exacerbation of depressive symptoms [11]. Genetic and longitudinal data also indicate that abnormal sleep duration—whether short or long—is linked to increased depression risk and adverse mental health trajectories [12–15].

Given that depression is also strongly associated with an increased mortality [16, 17], and that both abnormal sleep duration and depression exhibit similar non-linear associations with death risk, we hypothesize that depressive symptoms may mediate the relationship between sleep duration and mortality [13, 18]. This conceptual model is supported by evidence that depressive symptoms affect multiple biological and behavioral pathways—including systemic inflammation [19], neuroendocrine dysregulation [20, 21], and reduced treatment adherence [22]—that are directly linked to premature death. Importantly, while the relationship between sleep and depression is bidirectional, longitudinal evidence suggests that sleep disturbances frequently precede the onset or worsening of depressive symptoms, particularly among older adults and those with chronic conditions [23–25]. This temporal ordering supports modeling sleep duration as the exposure and depressive symptoms as the mediator.

Although current academic discourse exploring the relationship between sleep duration and depression or mortality does not explicitly distinguish between objective and subjective sleep duration, some studies have shown low correlations between self-reported sleep duration and objectively measured sleep duration [26, 27]. However, there are no studies that quantify and compare the effects of objective and subjective sleep duration on mortality and the role that depressive symptoms may play in these relationships.

Structural equation modeling (SEM) is a sophisticated statistical framework that calculates path coefficients, determines the direction and degree of mediators, which effectively elucidates the role mediators play between independent and dependent variables. It combines confirmatory factor analysis with multiple regression paths, which can skillfully handle latent variables and measurement errors, and is a powerful tool for exploring the aforementioned issues [28, 29].

Therefore, this study used data from the National Health and Nutrition Examination Survey (NHANES) and applied SEM to assess and quantify the influence of the Patient Health Questionnaire (PHQ-9) score on the relationship between objective and subjective sleep durations and all-cause mortality. This study aims to investigate whether subjective and objective sleep duration differ in their associations with all-cause mortality, and whether depressive symptoms mediate these associations, thereby extending the existing literature and informing future clinical and public health strategies.

## Methods

### Study design and population

NHANES is a nationally representative health survey conducted by the National Center for Health Statistics (NCHS) of the Centers for Disease Control and Prevention (CDC). NHANES collects data on a 2-year cycle and uses a complex multistage probability sampling design to select a representative sample from the civilian, non-institutionalized US population. The protocol has been approved by the NCHS Research Ethics Review Board (Continuation of Protocol #2011-17 http://www.cdc.gov/nchs/nhanes/irba98.htm) and all participants provided written informed consent.

The initial sample size was 19,931 participants in the 2011–2014 NHANES dataset. After excluding participants aged < 18 years (*n* = 7954), physical activity monitor (PAM) data ineligible (*n* = 4113), missing follow-up information (*n* = 16), and missing self-reported sleep duration data (*n* = 10), the final analysis included 7838 adults with complete baseline data on objective and subjective sleep duration.

### Data collection

Participants completed a detailed questionnaire at home, which was administered by trained researchers and covered demographic characteristics, healthy lifestyle (smoking, alcohol use, physical activity, sleep disorders), mental health (including the PHQ-9), diet and medical conditions. Following the interview, participants visited Mobile Health Screening Centre (MHC) for a series of anthropometric and physiological assessments, including PAM.

### Assessment of depression

Depressive symptoms were measured using the PHQ-9, a nine-item screening instrument that asked questions about the frequency of symptoms of depression over the past 2 weeks: (1) Have little interest in doing things; (2) Feeling down, depressed, or hopeless; (3) Trouble sleeping or sleeping too much; (4) Feeling tired or having little energy; (5) Poor appetite or overeating; (6) Feeling bad about yourself; (7) Trouble concentrating on things; (8) Moving or speaking slowly or too fast; (9) Thought you would be better off dead. Response categories for the nine-item instrument “not at all,” “several days,” “more than half the days” and “nearly every day” were given a point ranging from 0 to 3 [30]. Depressive symptoms in this study were quantified as the total PHQ-9 score.

### Assessment of sleep duration

Subjective (self-reported) sleep duration was determined by participants’ responses to the question, “How much sleep do you get (hours)?” from the Sleep Disorders Questionnaire. Responses ranged from 2 to 11 h, while values of 12 h or more were uniformly recoded as 12 h in accordance with NHANES protocols. As extreme values above 12 h/day are rare and may reflect reporting errors or atypical conditions (e.g., hospitalization), recoding can help minimize the influence of outliers and reduce data skewness. Objective sleep duration was measured using the ActiGraph model GT3X + PAM (ActiGraph of Pensacola, FL). NHANES participants were asked to wear the PAM for seven consecutive days to gather seven segments of accurate 24-hour activity data from each individual [31]. Data quality reviews processed signals deemed unlikely to result from human movement and flagged the minute as “invalid.” The minute summary record contained both valid measured minutes (classified as taken during “wake”, “sleep”, “non-wear”, or “unknown” time period) and identified invalid measured minutes. We excluded ineligible PAM data with “non-wear”, “unknown”, “invalid” minutes > 60 min/day, or valid “wake”/“sleep” wear minutes < 120 min/day. The “sleep” wear minutes for the remaining data for each individual were averaged to calculate the objective sleep duration. These criteria were designed to exclude unreliable recordings to improve the accuracy of sleep duration estimation.

As the recommended sleep duration is 7–9 h, sleep deprivation was defined as sleeping < 6 h/day [32, 33] and sleep overload as sleeping ≥ 10 h/day [8, 18].

### Ascertainment of outcome

The primary outcome was all-cause mortality, and the secondary outcome was fatal major adverse cardiovascular events (MACE), which included deaths caused by heart disease (ICD-10 codes I00-I09, I11, I13, I20-I51) and cerebrovascular diseases (ICD-10 codes I60-I69). Data were sourced from the NHANES public-use linked mortality file, connected to the National Death Index (NDI). All participants were linked to mortality data up to December 31, 2019.

### Statistical analysis

#### Descriptive statistics

We applied NHANES-recommended weights to adjust for the oversampling of specific demographic groups. Continuous variables are presented as means (standard deviation) and categorical variables are presented as counts (percentages). Continuous variables were compared by the two independent samples t-test or ANOVA, and categorical variables were compared by the χ^2^ test.

### Regression analysis

The relationship between objective and subjective sleep duration and PHQ-9 score was assessed using linear regression. The relationship between objective and subjective sleep duration, PHQ-9, and all-cause mortality was assessed using multifactor adjusted Cox proportional hazards models to calculate hazard ratio (HR) and 95% confidence interval (CI). Model 1 was adjusted for age and sex, and Model 2 was further adjusted for race, education level, poverty/income ratio, current smoking, body mass index (BMI), hypertension, and diabetes. In the fully adjusted model, restricted cubic spline (RCS) analysis was used to visualize potential linear or non-linear relationships between objective and subjective sleep duration (continuous) and mortality risk.

Considering the U-shaped relationship between sleep duration and mortality risk observed in the restricted cubic spline analysis, we used the mean of the sleep duration (7.17 h/day for objective Sleep duration and 6.67 h/day for subjective sleep duration) least associated with risk of death as the cut-off point (7 h/day), and conducted regression analyses separately for those with sleep duration < 7 h/day and those with sleep duration ≥ 7 h/day. In addition, we assessed the mortality risk for sleep deprivation (< 6 h/day) and sleep overload (≥ 10 h/day) separately, using a sleep duration of 6–10 h/day as a reference. We also performed Cox regressions for the interaction subgroups of objective and/or subjective sleep deprivation (< 6 h/day) to compare the significance of objective and subjective sleep deprivation in predicting risk of mortality.

### Structural equation modelling

Variables associated with both sleep duration and all-cause mortality were included in the SEM. By including a third hypothetical variable (PHQ-9 score), the SEM allowed for the identification and explanation of the mechanisms underlying the relationship between the independent variable (sleep duration) and the dependent variable (all-cause mortality) and quantify the percentage of mediating effect in total effect. The model was also adjusted for age, sex, race, education level, poverty/income ratio, current smoking, BMI, hypertension, and diabetes.

Different paths were created in this model: Path a, representing the effect of sleep duration on PHQ-9 score; Path b, representing the effect of PHQ-9 score on all-cause mortality; Path a*b (known as the indirect effect), which represents the mediated effect of sleep duration on all-cause mortality through the mediator; Path c, representing the total effect of sleep duration on all-cause mortality; and Path c’, which represents the remaining effect of sleep duration on all-cause mortality not mediated by the mediator.

Considering the U-shaped relationship between sleep duration and mortality risk, we assessed separately using the mean of sleep duration (7 h/day) as the cut-off. The same analyses were repeated in models with fatal MACE as the dependent variable.

All statistical analyses were conducted using the “lavaan” (version 0.6–17) packages in the R software (version 4.3.2; R Foundation for Statistical Computing, Vienna, Austria). Statistical significance was defined as *P* < 0.05.

## Results

### Participant characteristics and prevalence

The study comprised 7838 participants, including 4392 women (55.96%) and 3446 men (44.04%), with an average age of 46.51 (0.46) years. Compared to participants with a sleep duration of 6–10 h/day, participants who reported sleep deprivation (< 6 h/day) or sleep overload (≥ 10 h/day) had lower rates of current drinking, higher PHQ-9 scores, lower coffee consumption, were more likely to have diabetes, hypertension, whether based on objective or subjective sleep duration. Baseline characteristics based on objective and subjective sleep duration status subgroups are shown in Table 1.

**Table 1:** Baseline characteristics based on objective and subjective sleep duration status subgroups

Characteristic	Total	Sleep Duration (obj.)			Sleep Duration (subj.)		
		< 6 h/d	6 ~ < 10 h/d	≥ 10 h/d	< 6 h/d	6 ~ < 10 h/d	≥ 10 h/d
	*n* = 7838	*n* = 897	*n* = 6261	*n* = 680	*n* = 1169	*n* = 6450	*n* = 219
Age, years, mean (SD)	46.51(0.46)	43.55(0.78)	46.27(0.48)	51.88(1.21)	45.52(0.83)	46.64(0.48)	47.19(1.88)
Women, *n* (%)	4392(55.96)	375(40.97)	3621(57.57)	396(56.79)	654(56.45)	3610(55.75)	128(60.82)
Race/Ethnicity, *n* (%)							
Non-Hispanic Black	1831(11.32)	350(23.52)	1342(10.14)	139(9.32)	408(19.47)	1368(10.00)	55(14.48)
Non-Hispanic White	3080(65.87)	200(44.58)	2526(67.36)	354(74.69)	370(56.44)	2618(67.47)	92(59.97)
Mexican American	1018(9.25)	121(13.30)	836(9.18)	61(5.53)	121(8.50)	865(9.27)	32(12.61)
Other Hispanic	800(6.40)	108(9.62)	635(6.22)	57(4.67)	136(8.33)	642(6.11)	22(6.62)
Other Race	1109(7.16)	118(8.99)	922(7.09)	69(5.78)	134(7.26)	957(7.16)	18(6.32)
Education level, *n* (%)							
High school graduate or higher	5724(80.92)	649(77.57)	4592(81.56)	483(78.50)	853(78.81)	4748(81.72)	123(63.37)
Less than high school	2114(19.08)	248(22.43)	1669(18.44)	197(21.50)	316(21.19)	1702(18.28)	96(36.63)
Poverty/income ratio, *n* (%)							
≥ 300%	2541(44.04)	272(36.62)	2093(45.78)	176(35.60)	290(29.18)	2214(46.89)	37(22.48)
< 300%	5297(55.96)	625(63.38)	4168(54.22)	504(64.40)	879(70.82)	4236(53.11)	182(77.52)
Current smoking, *n* (%)	3195(42.36)	371(41.66)	2509(41.86)	315(47.75)	561(50.85)	2537(40.92)	97(47.67)
Current drinking, *n* (%)	4863(68.65)	570(66.93)	3924(69.58)	369(61.77)	711(63.94)	4041(69.75)	111(54.52)
BMI, kg/m^2^, mean (SD)	28.73(0.14)	30.90(0.45)	28.38(0.14)	29.78(0.37)	30.26(0.26)	28.51(0.14)	28.40(0.52)
Physical activity, min/w, mean (SD)	848.29(27.58)	990.83(76.42)	860.82(28.31)	578.27(67.17)	1118.65(82.36)	812.00(28.21)	675.60(105.41)
PHQ-9 score, mean (SD)	2.92(0.08)	3.05(0.23)	2.78(0.08)	4.12(0.23)	4.82(0.25)	2.59(0.07)	4.55(0.48)
Alcohol, cup, mean (SD)	1.79(0.05)	2.12(0.11)	1.76(0.05)	1.69(0.12)	1.88(0.10)	1.78(0.05)	1.58(0.21)
Coffee, cup, mean (SD)	1.61(0.05)	1.31(0.20)	1.66(0.06)	1.47(0.13)	1.53(0.11)	1.64(0.06)	1.10(0.17)
Diabetes, *n* (%)	1206(11.78)	147(15.22)	907(10.85)	152(16.84)	211(13.93)	952(11.36)	43(15.11)
Hypertension, *n* (%)	3900(47.74)	475(51.02)	3005(46.06)	420(60.05)	663(52.96)	3116(46.83)	121(51.96)
Fatal MACE, *n* (%)	182(1.78)	14(1.30)	120(1.45)	48(5.41)	24(1.80)	138(1.60)	20(8.33)
All-cause mortality, *n* (%)	582(6.28)	50(5.84)	385(5.05)	147(18.28)	94(7.83)	443(5.71)	45(18.40)
Sleep Related							
Sleep duration (obj.), hour/d, mean (SD)	7.75(0.03)	5.34(0.03)	7.67(0.02)	11.16(0.03)	7.44(0.07)	7.77(0.03)	8.97(0.16)
Sleep duration (subj.), hour/d, mean (SD)	6.92(0.02)	6.32(0.05)	6.95(0.03)	7.29(0.09)	4.57(0.02)	7.18(0.02)	10.39(0.06)
Sleep duration (obj.) < 6 h/d, *n* (%)	897(9.33)	897(100.00)	0(0.00)	0(0.00)	239(17.24)	648(8.28)	10(4.09)
Sleep duration (subj.) < 6 h/d, *n* (%)	1169(12.87)	239(23.77)	844(11.70)	86(12.22)	1169(100.00)	0(0.00)	0(0.00)

*Abbreviations*: *BMI* body mass index,* PHQ-9* Patient Health Questionnaire, *MACE* major adverse cardiovascular events

During a median follow-up period of 6.83 years, we observed 582 deaths among the participants, including 182 cases of fatal MACE. These individuals were older, more probable current smokers, had less physical activity time, higher PHQ-9 scores, and were more likely to have diabetes and hypertension. They had a higher prevalence of subjective sleep deprivation (< 6 h/day), although their average objective and subjective sleep duration were longer. Baseline characteristics according to survival status are detailed in Supplementary Table 1.

### Regression analysis of sleep duration, PHQ-9 score and all-cause mortality

**Fig. 1:**
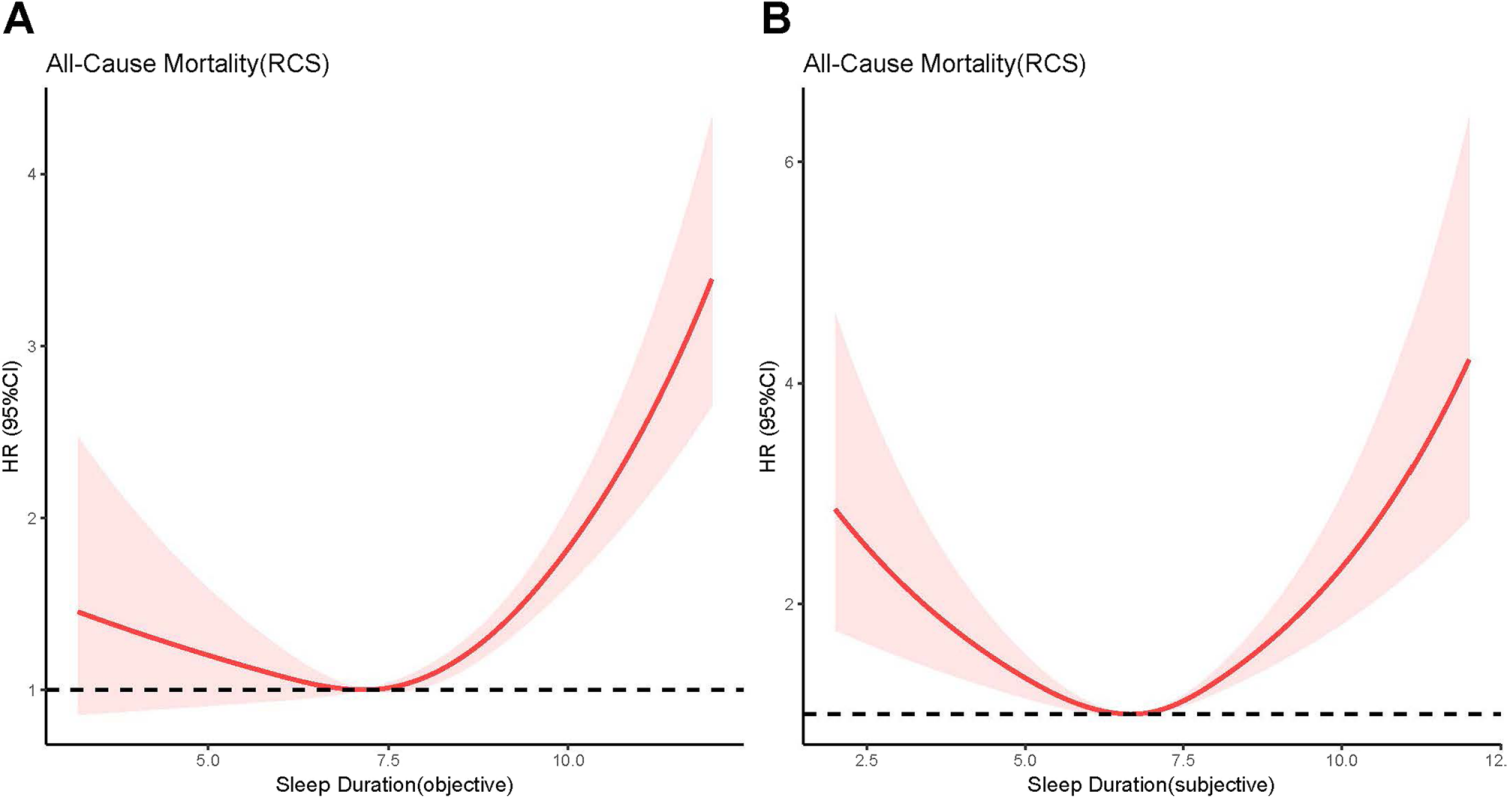
The Restricted Cubic Spline Analysis of Sleep Duration and All-Cause Mortality Risk. (**A**) Objective Sleep Duration (Ref =7.17, *P* for nonlinear<0.001). (**B**) Subjective Sleep Duration (Ref =6.67, *P* for nonlinear <0.001)

Cox regression models showed that each additional hour of objective sleep duration was associated with a higher risk of all-cause mortality (HR: 1.189, 95%CI: 1.122, 1.261, *P* < 0.001) but similar results were not observed for subjective sleep duration. When sleep duration of 6–10 h/day was used as reference, objective and subjective sleep deprivation (obj. HR: 1.724, 95%CI: 1.085, 2.740, *P* = 0.021; subj. HR: 1.560, 95%CI: 1.140, 2.133, *P* = 0.021) and sleep overload (obj. HR: 2.464, 95%CI: 1.991, 3.049, *P* < 0.001; subj. HR: 2.277, 95%CI: 1.617, 3.207, *P* < 0.001) both increased the risk of all-cause mortality (Table 2). Compared with participants with both objective and subjective sleep duration ≥ 6 h/day, only objective sleep duration < 6 h/day did not increase the all-cause mortality risk, whereas only subjective sleep duration < 6 h/day increased the risk of all-cause mortality by 38.5% (HR: 1.385, 95%CI: 1.042, 1.839, *P* = 0.025, Table 2). The RCS curves demonstrated a J-shaped relationship between objective sleep duration and the all-cause mortality risk (*P* for nonlinear < 0.001, Fig. 1A), and a U-shaped relationship between subjective sleep duration and the all-cause mortality risk (*P* for nonlinear < 0.001, Fig. 1B). A J-shaped association implies an increased risk at both very short and very long durations with a steeper rise on the long-duration side, while a U-shaped curve indicates symmetric risk at both extremes of duration.

**Table 2:** Regression analysis of sleep duration, PHQ-9 score and All-Cause mortality

Paths			Model 1		Model 2	
Sleep Duration → All-Cause Mortality			HR (95%CI)	*P* value	HR (95%CI)	*P* value
Sleep duration (obj.), hour/d			1.212(1.147,1.280)	< 0.001	1.189(1.122,1.261)	< 0.001
Sleep duration (subj.), hour/d			1.023(0.938,1.115)	0.604	1.038(0.946,1.140)	0.429
Objective Sleep Duration Group	6 ~ < 10 h/d		1[Ref]		1[Ref]	
	< 6 h/d		1.709(1.090,2.678)	0.019	1.724(1.085,2.740)	0.021
	≥ 10 h/d		2.547(2.106,3.080)	< 0.001	2.464(1.991,3.049)	< 0.001
Subjective Sleep Duration Group	6 ~ < 10 h/d		1[Ref]		1[Ref]	
	< 6 h/d		1.784(1.372,2.320)	< 0.001	1.560(1.140,2.133)	0.005
	≥ 10 h/d		2.545(1.827,3.544)	< 0.001	2.277(1.617,3.207)	< 0.001
Objective and Subjective Sleep Duration Group	obj. ≥6 h/d	subj. ≥6 h/d	1[Ref]		1[Ref]	
	obj. <6 h/d	subj. ≥6 h/d	1.274(0.694,2.339)	0.436	1.287(0.690,2.401)	0.428
	obj. ≥6 h/d	subj. <6 h/d	1.605(1.266,2.034)	< 0.001	1.385(1.042,1.839)	0.025
	obj. <6 h/d	subj. <6 h/d	2.578(1.202,5.529)	0.015	2.498(1.125,5.545)	0.024
Sleep Duration → PHQ-9 Score			*β* (95%CI)	*P* value	*β* (95%CI)	*P* value
Sleep duration (obj.), hour/d			0.177(0.089, 0.264)	< 0.001	0.161(0.080, 0.241)	< 0.001
Sleep duration (subj.), hour/d			−0.397(−0.517, −0.276)	< 0.001	−0.343(−0.466, −0.221)	< 0.001
PHQ-9 Score → All-Cause Mortality			HR (95%CI)	*P* value	HR (95%CI)	*P* value
PHQ-9 score			1.062(1.038,1.087)	< 0.001	1.051(1.023,1.079)	< 0.001

Adjusted for age, sex, race, education level, poverty/income ratio, current smoking, BMI, hypertension, and diabetes

*Abbreviations*: *BMI* body mass index, *PHQ-9* Patient Health Questionnaire

Linear regression modelling showed that each additional hour of objective sleep duration was associated with higher PHQ-9 scores (*β*: 0.161, 95%CI: 0.080, 0.241, *P* < 0.001), whereas each one-hour reduction in subjective sleep duration was associated with higher PHQ-9 scores (*β*: −0.342, 95%CI: −0.466, −0.221, *P* < 0.001). Meanwhile, increased PHQ-9 scores were associated with an increased risk of all-cause mortality (HR: 1.051, 95%CI: 1.023, 1.079, *P* < 0.001, Table 2).

### Regression analysis in objective sleep duration < 7 h/day

When objective sleep duration < 7 h/day, regression analyses showed that objective sleep duration and PHQ-9 score were not associated with the all-cause mortality in the fully adjusted model (Table 3).

**Table 3:** Regression analysis of sleep duration, PHQ-9 score and All-Cause mortality according to sleep duration status

Paths	Model 1		Model 2	
Sleep Duration < 7 h/day				
Sleep Duration → All-Cause Mortality	HR (95%CI)	*P* value	HR (95%CI)	*P* value
Sleep duration (obj.), hour/d	0.758(0.587,0.979)	0.034	0.796(0.613,1.034)	0.088
Sleep duration (subj.), hour/d	0.751(0.669,0.843)	< 0.001	0.799(0.686,0.931)	0.004
Sleep Duration → PHQ-9 Score	***β*** (95%CI)	*P* value	***β*** (95%CI)	*P* value
Sleep duration (obj.), hour/d	−0.135(−0.410, 0.139)	0.322	−0.008(−0.025, 0.008)	0.832
Sleep duration (subj.), hour/d	−1.128(−1.431, −0.824)	< 0.001	−0.938(−1.242, −0.634)	< 0.001
PHQ-9 Score → All-Cause Mortality	HR (95%CI)	*P* value	HR (95%CI)	*P* value
PHQ-9 score (obj.<7 h/d)	1.044(1.003,1.087)	0.033	1.004(0.952,1.059)	0.874
PHQ-9 score (subj. <7 h/d)	1.055(1.029,1.083)	< 0.001	1.055(1.027,1.083)	< 0.001
Sleep Duration ≥ 7 h/day				
Sleep Duration → All-Cause Mortality	HR (95%CI)	*P* value	HR (95%CI)	*P* value
Sleep duration (obj.), hour/d	1.347(1.248,1.454)	< 0.001	1.335(1.233,1.445)	< 0.001
Sleep duration (subj.), hour/d	1.308(1.191,1.438)	< 0.001	1.269(1.144,1.408)	< 0.001
Sleep Duration → PHQ-9 Score	***β*** (95%CI)	*P* value	***β*** (95%CI)	*P* value
Sleep duration (obj.), hour/d	0.394(0.260, 0.527)	< 0.001	0.308(0.181, 0.435)	< 0.001
Sleep duration (subj.), hour/d	0.450(0.300, 0.599)	< 0.001	0.336(0.168, 0.503)	< 0.001
PHQ-9 Score → All-Cause Mortality	HR (95%CI)	*P* value	HR (95%CI)	*P* value
PHQ-9 score (obj.≥7 h/d)	1.067(1.037,1.097)	< 0.001	1.063(1.031,1.097)	< 0.001
PHQ-9 score (subj.≥7 h/d)	1.069(1.031,1.108)	< 0.001	1.050(1.006,1.096)	0.027

Adjusted for age, sex, race, education level, poverty/income ratio, current smoking, BMI, hypertension, and diabetes

*Abbreviations*: *BMI* body mass index, *PHQ-9* Patient Health Questionnaire

### Regression analysis in subjective sleep duration < 7 h/day

Subjective sleep duration increases were associated with reduced risk of all-cause mortality (HR: 0.799, 95%CI: 0.686, 0.931, *P* = 0.004), subjective sleep duration negatively correlated with PHQ-9 scores (*β*: −0.938, 95%CI: −1.242, −0.634, *P* < 0.001), and PHQ-9 scores were associated with increased risk of all-cause mortality (HR: 1.055, 95%CI: 1.027, 1.083, *P* < 0.001, Table 3).

### Regression analysis in objective sleep duration ≥ 7 h/day

When objective sleep duration ≥ 7 h/day, objective sleep duration increases were associated with increased risk of all-cause mortality (HR: 1.335, 95%CI: 1.233, 1.445, *P* < 0.001), objective sleep duration positively correlated with PHQ-9 scores (*β*: 0.308, 95%CI: 0.181, 0.435, *P* < 0.001), and PHQ-9 scores were associated with increased risk of all-cause mortality (HR: 1.063, 95%CI: 1.031, 1.097, *P* < 0.001, Table 3).

### Regression analysis in subjective sleep duration ≥ 7 h/day

Subjective sleep duration increases were associated with increased risk of all-cause mortality (HR: 1.269, 95%CI: 1.144, 1,408, *P* < 0.001), subjective sleep duration positively correlated with PHQ-9 scores (*β*: 0.336, 95%CI: 0.168, 0.503, *P* < 0.001), and PHQ-9 scores were associated with increased risk of all-cause mortality (HR: 1.050, 95%CI: 1.006, 1.096, *P =* 0.027, Table 3).

### Structural equation modelling

We constructed an SEM of sleep duration and all-cause mortality, with PHQ-9 scores as the mediator variable.

**Fig. 2:**
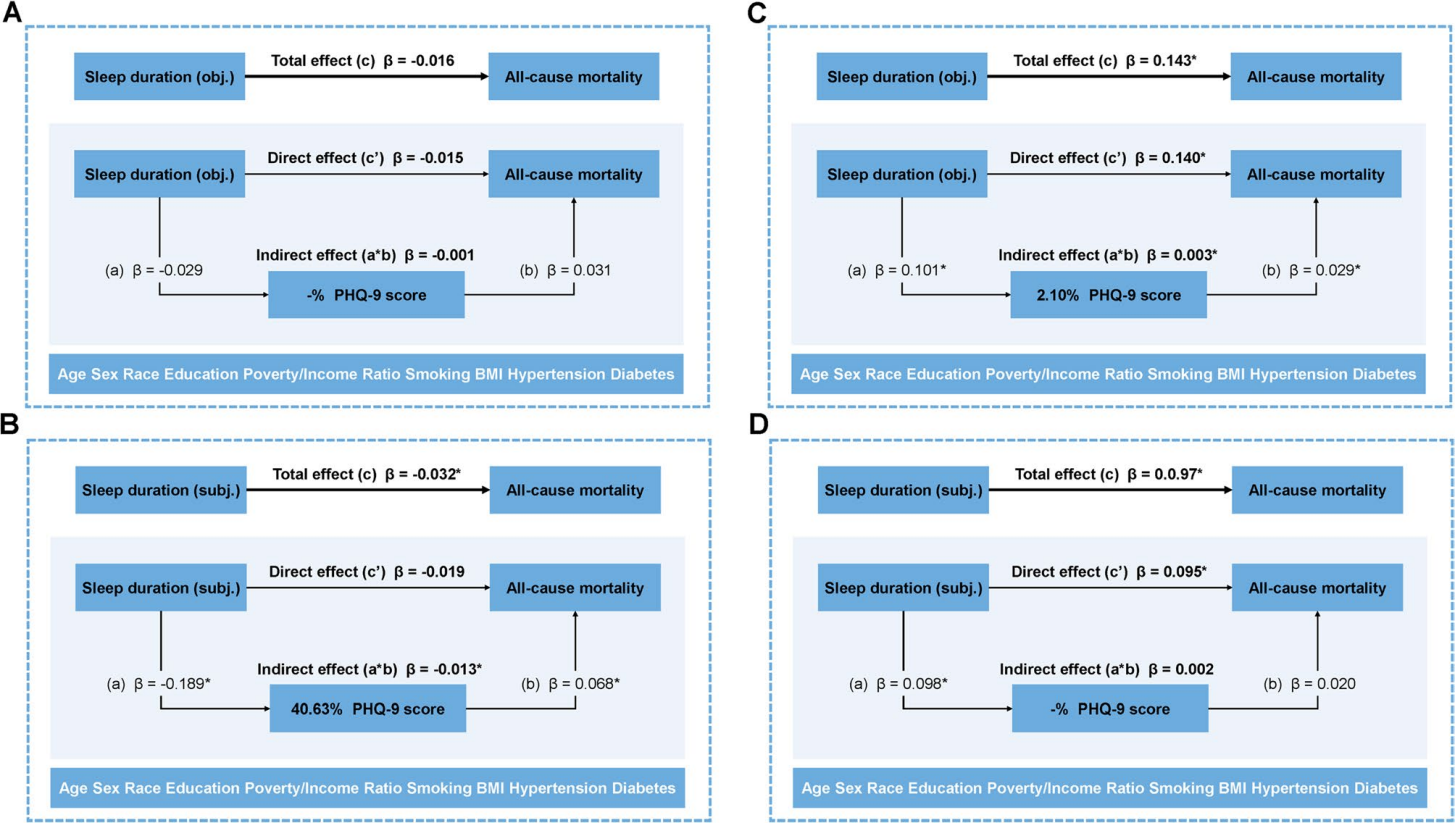
Structural Equation Modelling of All-Cause Mortality. (**A**) Objective Sleep Duration <7 hours/day; (**B**) Subjective Sleep Duration <7 hours/day; (**C**) Objective Sleep Duration ≥7 hours/day; (**D**) Subjective Sleep Duration ≥7 hours/day; Abbreviations:BMI, body mass index; PHQ-9, Patient Health Questionnaire. Adjusted for age, sex, race, education level, poverty/income ratio, current smoking, BMI, hypertension, and diabetes

The results showed that the direct effects or indirect effects of objective sleep duration on all-cause mortality were not significant when sleep duration was < 7 h/day (Fig. 2A). The total effect (path c) of subjective sleep duration on all-cause mortality was − 0.032 (*P* = 0.048), the direct effect (path c’) was − 0.019 (*P* = 0.333), and the indirect effect (path a*b) was − 0.013 (*P* = 0.003), which accounted for (a*b/c) 40.63% of the total effect (Fig. 2B, Supplementary Table 2).

When sleep duration was ≥ 7 h/day, the total effect (path c) of objective sleep duration on all-cause mortality was 0.143 (*P* < 0.001), the direct effect (path c’) was 0.140 (*P* < 0.001), and the indirect effect (path a*b) was 0.003 (*P* = 0.028), which accounted for (a*b/c) 2.10% of the total effect (Fig. 2C). The total effect (path c) of subjective sleep duration on all-cause mortality was 0.097 (*P* < 0.001), the direct effect (path c’) was 0.095 (*P* < 0.001), and the indirect effect (path a*b) was 0.002, which was not significant (*P* = 0.097, Fig. 2D, Supplementary Table 3).

### Sensitivity analysis

Regression analyses of sleep duration, PHQ-9 score and fatal MACE showed that when sleep duration of 6–10 h/day was used as reference, only sleep overload (obj. HR: 2.184, 95%CI: 1.410, 3.385, *P* < 0.001; subj. HR: 2.959, 95%CI: 1.716, 5.100, *P* < 0.001) increased the risk of fatal MACE (Supplementary Table 4).The RCS curves showed a J-shaped relationship between objective sleep duration and fatal MACE risk (*P* for nonlinear = 0.006, Supplementary Fig. 1 A) and a U-shaped relationship between subjective sleep duration and fatal MACE risk (*P* for nonlinear < 0.001, Supplementary Fig. 1B).

The SEM with fatal MACE as the dependent variable showed that PHQ-9 mediated the effect only in participants with subjective sleep duration < 7 h/day. When sleep duration was < 7 h/day, the total effect (path c) of subjective sleep duration on fatal MACE was − 0.016 (*P* = 0.443), the direct effect (path c’) was − 0.008 (*P* = 0.704), and the indirect effect (path a*b) was − 0.009 (*P* = 0.044), which accounted for (a*b/c) 56.25% of the total effect (Supplementary Table 5).

In addition, we observed a direct effect (path c’) of both subjective and objective sleep duration on fatal MACE when sleep duration was ≥ 7 h/day (obj. Standardized *β* = 0.087, *P* < 0.001; subj. Standardized *β* = 0.085, *P* < 0.001, Supplementary Table 6).

## Discussion

Using SEM as analysis tool, this study disentangles and reveals the relationship between sleep duration and mortality in more depth and locate the role that depressive symptoms play in it. Our study found a J-shaped relationship between objective sleep duration and all-cause mortality risk whereas subjective sleep duration showed a U-shaped relationship. Depressive symptom primarily mediated the relationship between subjective sleep deprivation and increased all-cause mortality risk, with a mediation rate of approximately 40%, and also mediated the relationship between objective sleep overload and all-cause mortality to a lesser extent.

There is a close bidirectional relationship between sleep disorders and depression. Approximately 80-90% of patients with depression experience some form of sleep disorder [34], and sleep disorders are closely associated with subsequent depression risk in both cross-sectional and longitudinal studies [10]. Multiple studies have further validated this relationship through neuroimaging [35–37], electroencephalography [38–40], pharmacological interventions [41–43], cognitive behavioral therapy [44]. Due to the high overlap between sleep disorders and depression, it is often mistakenly believed that sleep problems are merely a secondary cause of depression [1]. For example, research reports a U-shaped correlation between sleep duration and mortality risk [3, 5, 6, 45], and depression is often one of the potential mechanisms’ researchers speculate may contribute to mortality. Currently, sleep problems are increasingly recognized as an independent key factor affecting prognosis [1], but the extent to which the impact of sleep problems on mortality is mediated by depression remains unknown. This study employs SEM to examine the relationship between sleep duration— an important dimension of sleep—and mortality, while quantifying the mediating role of depression symptoms in this relationship.

A large number of studies have shown a U-shaped relationship between sleep duration and mortality. However, most studies have used self-reported sleep duration metrics, and this subjective sleep duration correlates poorly with objectively measured sleep duration [18]. In particular, the presence of psychiatric disorders often leads to patients’ subjective misjudgment of their sleep status, so objective sleep monitoring is recommended [26, 46]. Sleep duration can be estimated using two main objective methods: polysomnography and actigraphy. Polysomnography, which measures sleep through electrical brain activity, is often considered the “gold standard” for defining sleep [47]. However, it is not ideal for determining typical sleep duration in population studies due to its invasive nature and the potential alteration of sleep patterns caused by the unfamiliar procedure. Although it can also be adapted for home use [48]. In contrast, actigraphy has been increasingly used in epidemiological studies [49, 50]. Research shows that polysomnography and actigraphy generally provide highly correlated sleep duration estimates, with an average correlation of about 90% [51]. Some studies have compared self-reported sleep duration with physiological monitoring results. For instance, in older adults, self-reported sleep duration averaged about one hour longer than polysomnography, with a correlation of just 0.18 [27]. Similarly, secondary analyses from the CARDIA study reported a correlation of 0.47 between actigraphy and self-report, with self-reported duration averaging 48 min longer [52]. In addition, individuals with risk factors for mortality, including depressive symptoms, tend to report shorter sleep duration for the same number of hours of sleep. This means that reporting bias may result in an association between reported subjective sleep deprivation and mortality even if there is no association between objective sleep duration and mortality [52]. This may partly explain our findings: depressive symptoms mediated around 40% of the association between subjective sleep deprivation and all-cause mortality. Additionally, when participants were divided into four groups based on objective and/or subjective sleep deprivation (< 6 h/day), we found that participants with only subjective sleep deprivation showed a significant increase in mortality risk. In contrast, the increase in mortality risk for those with only objective sleep deprivation was not significant. This finding emphasizes that, while subjective sleep deprivation may not always be accompanied by objective sleep reduction, its combined effect with psychiatric factors is strongly linked to poor prognosis. Even if objective sleep monitoring does not indicate sleep deprivation, clinicians should take patients’ primary complaints of sleep deprivation seriously, stay alert to potential underlying sleep disorders, and proactively provide both sleep-related guidance and psychological support.

Frequently, the relationship between sleep deprivation and adverse health outcomes is of greater concern, while there is usually no consensus on whether sleep overload is associated with health risks, thus many times it is recommended to get 7 or more hours of sleep [2]. The present study found that the direct effect of sleep deprivation and increased risk of mortality was not significant, but rather that sleep overload reflected a more significant direct risk, with a mediating effect of depressive symptoms of only about 2%. This is reflected in previous studies, that is, multiple meta-analyses also revealed a J-shaped relationship between sleep duration and risk of all-cause mortality [4–6]. The mechanisms underlying the association between sleep overload and all-cause mortality are not fully understood. Some believe that sleep overload probably represents the confounding effects of subfertile states or uncontrolled chronic diseases [53]. For example, sleep affects the body through inflammatory processes. Concentrations of inflammatory markers (e.g., interleukin 6 and C-reactive protein) increase when sleep duration is excessive [54, 55]. In addition, long bedtime is associated with increased sleep fragmentation [56], which is thought to be associated with more severe atherosclerosis and subcortical meatus infarcts. These are independent risk factors for cardiovascular disease and multiple medical comorbidities [57]. Finally, sleep overload has been associated with excessive fatigue, shortened physiological cycles based on circadian rhythms [45], low socioeconomic status, unemployment, low household income, low education, and other potential disease processes associated with mortality [58]. Further experimental studies are necessary to explore the potential effects of sleep overload on health outcomes. In particular, future research could incorporate cytokine profiling (e.g., interleukin-6, C-reactive protein) and polysomnographic assessments to more directly examine the biological and neurophysiological mechanisms underlying the observed associations. Investigating how alterations in sleep architecture or inflammatory status interact with depressive symptoms may help clarify their respective contributions to mortality risk, and whether interventions targeting sleep quality and mental health can mitigate these effects.

The present study has some limitations. Firstly, the objective sleep monitoring is only for 7 days, which may not be able to respond to some special situations and reflect a long-term sleep pattern well. Second, there are still many sleep components not considered, such as sleep quality and sleep regularity. Although sleep duration is the most commonly studied sleep indicator related to health [2]. Third, due to database limitations, there were some other confounders that were not considered, such as sleep apnea, physical activity, coffee, and alcohol use. We added some of the confounders to construct the SEM for sleep duration and mortality again, and again validated our findings (Supplementary Table 7). Fourth, objective sleep duration in this study was estimated using data from the ActiGraph GT3X+. Although actigraphy is a widely accepted tool in large-scale research, it detects movement rather than sleep directly, and may misclassify quiet wakefulness as sleep. This could lead to overestimation of true sleep duration and introduce measurement bias. Future studies incorporating light-sensing capabilities or polysomnographic validation may enhance the accuracy of objective sleep assessment. Finally, although we hypothesized a mediation pathway from sleep duration to depressive symptoms to mortality, the temporal sequence of data collection does not fully support causal inference. Specifically, depressive symptoms were assessed at baseline, whereas objective sleep duration was measured over a subsequent 7-day period. This time gap limits our ability to definitively determine the directionality of the association between sleep and depressive symptoms.

## Conclusion

In conclusion, our study found a J-shaped relationship between objective sleep duration and mortality risk and a U-shaped relationship between subjective sleep duration and mortality risk. Depressive symptoms mediated approximately 40% of the association between subjective sleep deprivation and mortality. These findings highlight the importance of recognizing the health risks associated with subjective sleep deprivation, particularly when co-occurring with depressive symptoms, and underscore the need for integrated clinical attention to both sleep disturbances and mental health.

## Supplementary Information

Supplementary Material 1. Supplementary Table 1. Baseline characteristics according to survival status. Supplementary Table 2. Structural Equation Modeling: Effects of PHQ-9 Score on Sleep Duration (<7 hours/day) and All-Cause Mortality. Supplementary Table 3. Structural Equation Modeling: Effects of PHQ-9 Score on Sleep Duration (≥7 hours/day) and All-Cause Mortality. Supplementary Table 4. Regression Analysis of Sleep Duration, PHQ-9 Score and Fatal Major Adverse Cardiovascular Events. Supplementary Table 5. Structural Equation Modeling: Effects of PHQ-9 Score on Sleep Duration (<7 hours/day) and Fatal MACE. Supplementary Table 6. Structural Equation Modeling: Effects of PHQ-9 Score on Sleep Duration (≥7 hours/day) and Fatal MACE. Supplementary Table 7. Structural Equation Modeling: Effects of PHQ-9 Score on Sleep Duration and All-Cause Mortality. Supplementary Figure 1. The Restricted Cubic Spline Analysis of Sleep Duration and Fatal Major Adverse Cardiovascular Events Risk. (A) Objective Sleep Duration (Ref =7.31, *P* for nonlinear =0.006). (B) Subjective Sleep Duration (Ref =6.52, *P* for nonlinear <0.001).

## Data Availability

The datasets supporting the conclusions of this article are available in the National Health and Nutrition Examination Survey repository https://www.cdc.gov/nchs/nhanes/.

## Abbreviations

BMI: body mass index
CDC: Centers for Disease Control and Prevention
CI: confidence interval
HR: hazard ratio
PAM: physical activity monitor
PHQ-9: Patient Health Questionnaire
RCS: restricted cubic spline
SEM: Structural equation modeling
MACE: major adverse cardiovascular events
MHC: Mobile Health Screening Centre
NCHS: National Center for Health Statistics
NDI: National Death Index
NHANES: National Health and Nutrition Examination Survey
