# Supplementary material for: Association among objective and subjective sleep duration, depressive symptoms and all-cause mortality: the pathways study
Source: BMC Psychiatry. 2025 Jul 29;25:735. doi: 10.1186/s12888-025-07181-9 (PMC12309052; doi:10.1186/s12888-025-07181-9)
Supplement: Supplementary file 1 — Supplementary Material 1. Supplementary Table 1. Baseline characteristics according to survival status. Supplementary Table 2. Structural Equation Modeling: Effects of PHQ-9 Score on Sleep Duration (<7 hours/day) and All-Cause Mortality. Supplementary Table 3. Structural Equation Modeling: Effects of PHQ-9 Score on Sleep Duration (≥7 hours/day) and All-Cause Mortality. Supplementary Table 4. Regression Analysis of Sleep Duration, PHQ-9 Score and Fatal Major Adverse Cardiovascular Events. Supplementary Table 5. Structural Equation Modeling: Effects of PHQ-9 Score on Sleep Duration (<7 hours/day) and Fatal MACE. Supplementary Table 6. Structural Equation Modeling: Effects of PHQ-9 Score on Sleep Duration (≥7 hours/day) and Fatal MACE. Supplementary Table 7. Structural Equation Modeling: Effects of PHQ-9 Score on Sleep Duration and All-Cause Mortality. Supplementary Figure 1. The Restricted Cubic Spline Analysis of Sleep Duration and Fatal Major Adverse Cardiovascular Events Risk. (A) Objective Sleep Duration (Ref =7.31, P for nonlinear =0.006). (B) Subjective Sleep Duration (Ref =6.52, P for nonlinear <0.001). [file 12888_2025_7181_MOESM1_ESM.docx]

**Supplementary Material**

**Association among Objective and Subjective Sleep Duration, Depressive Symptoms and All-Cause Mortality: The Pathways Study**

Yuan Zeng^1^**^*^**, Tanshu Liu^1^, Rui Qiu^1^, Qingqing Lian^1^

1 Department of Acupuncture and Moxibustion, Longyan First Affiliated Hospital of Fujian Medical University, Longyan, 364000, China

*** Correspondence:**

Yuan Zeng, MD

Department of Acupuncture and Moxibustion, Longyan First Affiliated Hospital of Fujian Medical University, Longyan, 364000, China

Tel: (+86) 05972958989/Fax: (+86) 05972292374

E-mail: 18020628091@163.com

**Supplementary Table 1. Baseline characteristics according to survival status.**

| **Characteristic** | **Total** | **Non-Mortality** | **All-Cause Mortality** | ***P* value** |
| --- | --- | --- | --- | --- |
|  | **n=7838** | **n=7256** | **n=582** |  |
| Age, years, mean (SD) | 46.51(0.46) | 45.16(0.49) | 66.57(0.82) | <0.001 |
| Women, n (%) | 4392(55.96) | 4087(55.95) | 305(56.00) | 0.980 |
| Race/Ethnicity, n (%) |  |  |  | <0.001 |
| Non-Hispanic Black | 1831(11.32) | 1694(11.34) | 137(11.05) |  |
| Non-Hispanic White | 3080(65.87) | 2745(65.03) | 335(78.39) |  |
| Mexican American | 1018(9.25) | 981(9.61) | 37(3.77) |  |
| Other Hispanic | 800(6.40) | 766(6.65) | 34(2.78) |  |
| Other Race | 1109(7.16) | 1070(7.37) | 39(4.01) |  |
| Education level, n (%) |  |  |  | 0.020 |
| High school graduate or higher | 5724(80.92) | 5342(81.29) | 382(75.37) |  |
| Less than high school | 2114(19.08) | 1914(18.71) | 200(24.63) |  |
| Poverty/income ratio, n (%) |  |  |  | <0.001 |
| ≥300% | 2541(44.04) | 2409(44.89) | 132(31.39) |  |
| <300% | 5297(55.96) | 4847(55.11) | 450(68.61) |  |
| Current smoking, n (%) | 3195(42.36) | 2863(41.28) | 332(58.37) | <0.001 |
| Current drinking, n (%) | 4863(68.65) | 4590(69.73) | 273(52.57) | <0.001 |
| BMI, kg/m^2^, mean (SD) | 28.73(0.14) | 28.75(0.15) | 28.53(0.41) | 0.620 |
| Physical activity, min/w, mean (SD) | 848.29(27.58) | 878.64(27.32) | 395.41(55.61) | <0.001 |
| PHQ-9 score, mean (SD) | 2.92(0.08) | 2.86(0.08) | 3.83(0.34) | 0.010 |
| Alcohol, cup, mean (SD) | 1.79(0.05) | 1.83(0.05) | 1.16(0.08) | <0.001 |
| Coffee, cup, mean (SD) | 1.61(0.05) | 1.59(0.06) | 1.85(0.17) | 0.190 |
| Diabetes, n (%) | 1206(11.78) | 1010(10.64) | 196(28.74) | <0.001 |
| Hypertension, n (%) | 3900(47.74) | 3431(45.63) | 469(79.24) | <0.001 |
| Fatal MACE, n (%) | 182(1.78) | 0(0.00) | 182(28.41) | <0.001 |
| **Sleep Related** |  |  |  |  |
| Sleep duration (obj.), hour/d, mean (SD) | 7.75(0.03) | 7.70(0.03) | 8.64(0.09) | <0.001 |
| Sleep duration (subj.), hour/d, mean (SD) | 6.92(0.02) | 6.90(0.03) | 7.14(0.08) | 0.010 |
| Sleep duration (obj.) <6 hours/d, n (%) | 897(9.33) | 847(9.38) | 50(8.68) | 0.700 |
| Sleep duration (subj.) <6 hours/d, n (%) | 1169(12.87) | 1075(12.66) | 94(16.04) | 0.070 |

**Abbreviations:** BMI, body mass index; PHQ-9, Patient Health Questionnaire; MACE, major adverse cardiovascular events.

**Supplementary Table 2. Structural Equation Modeling: Effects of PHQ-9 Score on Sleep Duration (<7** **hours/day) and All-Cause Mortality.**

| **Objective Sleep Duration** | | | | | | | | | |
| --- | --- | --- | --- | --- | --- | --- | --- | --- | --- |
| **Sleep duration effect on PHQ-9 score (**a: X→M**)** | | | | | **PHQ-9 score effect on All-cause mortality (**b: M→Y**)** | | | | |
| *β* | Lower | Upper | *P* value | Standardized *β* | *β* | Lower | Upper | *P* value | Standardized *β* |
| -0.164 | -0.380 | 0.052 | 0.137 | -0.029 | 0.002 | -0.001 | 0.004 | 0.168 | 0.031 |
| **Indirect Effect (**a*b: X→M→Y**)** | | | | | **Direct Effect (**c': X→Y adj M**)** | | | | |
| *β* | Lower | Upper | *P* value | Standardized *β* | *β* | Lower | Upper | *P* value | Standardized *β* |
| -0.000 | -0.001 | 0.000 | 0.299 | -0.001 | -0.004 | -0.015 | 0.007 | 0.455 | -0.015 |
| **Mediated (%) (**a*b/c**)** | | | | | **Total Effect (**c: X→Y**)** | | | | |
| - | | | | | *β* | Lower | Upper | *P* value | Standardized *β* |
|  |  |  |  |  | -0.005 | -0.016 | 0.007 | 0.428 | -0.016 |
| **Subjective Sleep Duration** | | | | | | | | | |
| **Sleep duration effect on PHQ-9 score (**a: X→M**)** | | | | | **PHQ-9 score effect on All-cause mortality (**b: M→Y**)** | | | | |
| *β* | Lower | Upper | *P* value | Standardized *β* | *β* | Lower | Upper | *P* value | Standardized *β* |
| -1.127 | -1.366 | -0.888 | <0.001 | -0.189 | 0.003 | 0.001 | 0.006 | 0.002 | 0.068 |
| **Indirect Effect (**a*b: X→M→Y**)** | | | | | **Direct Effect (**c': X→Y adj M**)** | | | | |
| *β* | Lower | Upper | *P* value | Standardized *β* | *β* | Lower | Upper | *P* value | Standardized *β* |
| -0.004 | -0.006 | -0.001 | 0.003 | -0.013 | -0.006 | -0.017 | 0.006 | 0.333 | -0.019 |
| **Mediated (%) (**a*b/c**)** | | | | | **Total Effect (**c: X→Y**)** | | | | |
| 40.63% | | | | | *β* | Lower | Upper | *P* value | Standardized *β* |
|  |  |  |  |  | -0.010 | -0.021 | -0.000 | 0.048 | -0.032 |

**Abbreviations:** BMI, body mass index; PHQ-9, Patient Health Questionnaire.

Adjusted for age, sex, race, education level, poverty/income ratio, current smoking, BMI, hypertension, and diabetes.

**Supplementary Table 3. Structural Equation Modeling: Effects of PHQ-9 Score on Sleep Duration (**≥**7** **hours/day) and All-Cause Mortality.**

| **Objective Sleep Duration** | | | | | | | | | |
| --- | --- | --- | --- | --- | --- | --- | --- | --- | --- |
| **Sleep duration effect on PHQ-9 score (**a: X→M**)** | | | | | **PHQ-9 score effect on All-cause mortality (**b: M→Y**)** | | | | |
| *β* | Lower | Upper | *P* value | Standardized *β* | *β* | Lower | Upper | *P* value | Standardized *β* |
| 0.343 | 0.237 | 0.449 | <0.001 | 0.101 | 0.002 | 0.001 | 0.004 | 0.023 | 0.029 |
| **Indirect Effect (**a*b: X→M→Y**)** | | | | | **Direct Effect (**c': X→Y adj M**)** | | | | |
| *β* | Lower | Upper | *P* value | Standardized *β* | *β* | Lower | Upper | *P* value | Standardized *β* |
| 0.001 | 0.000 | 0.001 | 0.028 | 0.003 | 0.031 | 0.024 | 0.037 | <0.001 | 0.140 |
| **Mediated (%) (**a*b/c**)** | | | | | **Total Effect (**c: X→Y**)** | | | | |
| 2.10% | | | | | *β* | Lower | Upper | *P* value | Standardized *β* |
|  |  |  |  |  | 0.031 | 0.024 | 0.038 | <0.001 | 0.143 |
| **Subjective Sleep Duration** | | | | | | | | | |
| **Sleep duration effect on PHQ-9 score (**a: X→M**)** | | | | | **PHQ-9 score effect on All-cause mortality (**b: M→Y**)** | | | | |
| *β* | Lower | Upper | *P* value | Standardized *β* | *β* | Lower | Upper | *P* value | Standardized *β* |
| 0.429 | 0.259 | 0.598 | <0.001 | 0.098 | 0.001 | -0.001 | 0.004 | 0.219 | 0.020 |
| **Indirect Effect (**a*b: X→M→Y**)** | | | | | **Direct Effect (**c': X→Y adj M**)** | | | | |
| *β* | Lower | Upper | *P* value | Standardized *β* | *β* | Lower | Upper | *P* value | Standardized *β* |
| 0.001 | 0.000 | 0.002 | 0.223 | 0.002 | 0.029 | 0.019 | 0.039 | <0.001 | 0.095 |
| **Mediated (%) (**a*b/c**)** | | | | | **Total Effect (**c: X→Y**)** | | | | |
| - | | | | | *β* | Lower | Upper | *P* value | Standardized *β* |
|  |  |  |  |  | 0.030 | 0.020 | 0.040 | <0.001 | 0.097 |

**Abbreviations:** BMI, body mass index; PHQ-9, Patient Health Questionnaire.

Adjusted for age, sex, race, education level, poverty/income ratio, current smoking, BMI, hypertension, and diabetes.


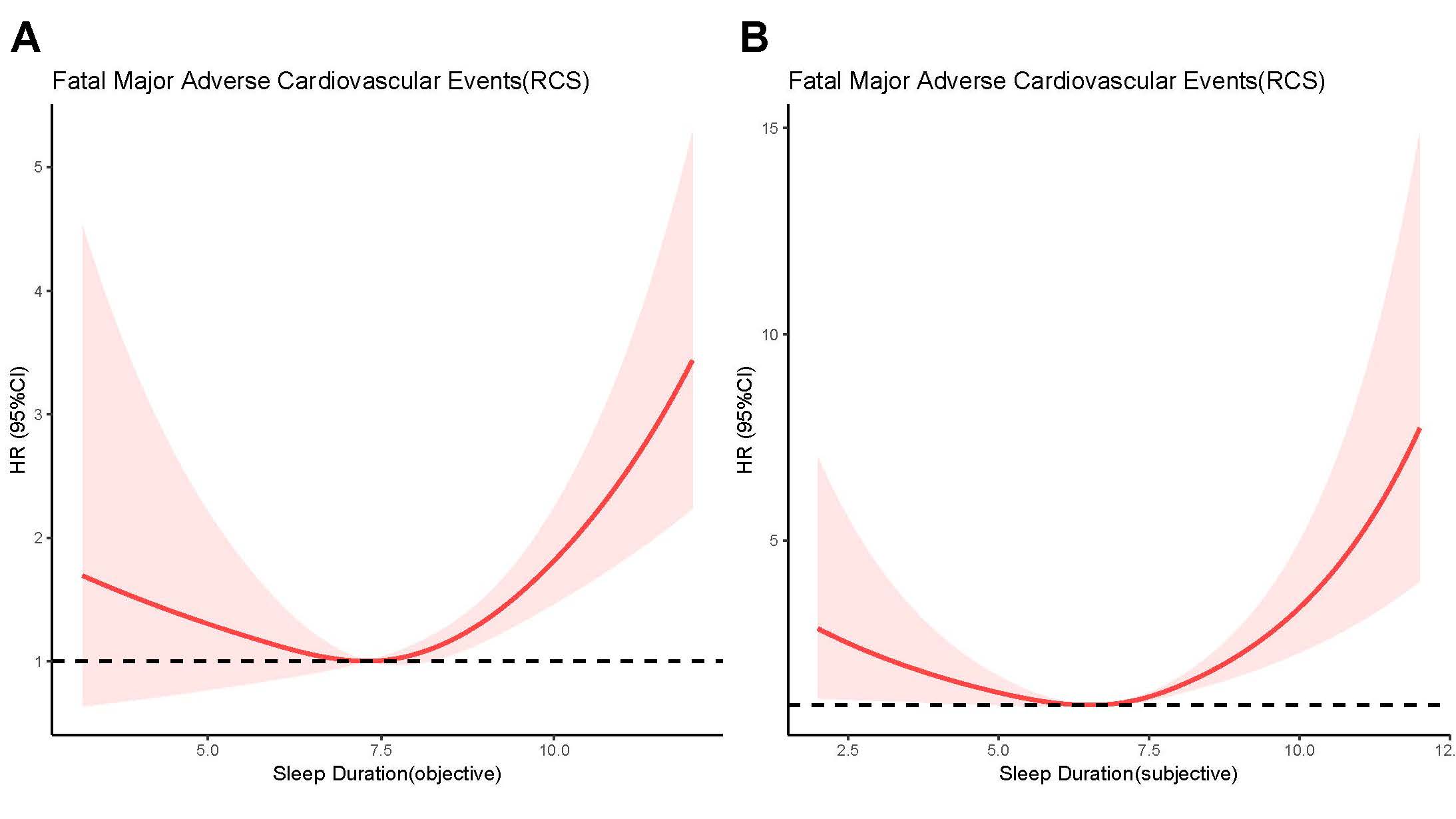


**Supplementary Figure 1**. **The Restricted Cubic Spline Analysis of Sleep Duration and Fatal Major Adverse Cardiovascular Events Risk**.

1. **Objective Sleep Duration** (Ref =7.31, *P* for nonlinear =0.006).
2. **Subjective Sleep Duration** (Ref =6.52, *P* for nonlinear <0.001).

**Supplementary Table 4. Regression Analysis of Sleep Duration, PHQ-9 Score and Fatal Major Adverse Cardiovascular Events.**

| **Paths** | | | **Model 1** | | **Model 2** | |
| --- | --- | --- | --- | --- | --- | --- |
| **Sleep Duration → Fatal MACE** | | | **HR (95%CI)** | ***P* value** | **HR (95%CI)** | ***P* value** |
| Sleep duration (obj.), hour/d | | | 1.266(1.142,1.404) | <0.001 | 1.243(1.119,1.380) | <0.001 |
| Sleep duration (subj.), hour/d | | | 1.162(1.008,1.340) | 0.039 | 1.191(1.046,1.355) | 0.008 |
| **Objective**  **Sleep Duration**  **Group** | 6~<10 hours/d | | 1[Ref] |  | 1[Ref] |  |
|  | <6 hours/d | | 1.498(0.720,3.113) | 0.279 | 1.274(0.597,2.722) | 0.531 |
|  | ≥10 hours/d | | 2.305(1.563,3.399) | <0.001 | 2.184(1.410,3.385) | <0.001 |
| **Subjective**  **Sleep Duration**  **Group** | 6~<10 hours/d | | 1[Ref] |  | 1[Ref] |  |
|  | <6 hours/d | | 1.569(1.108,2.223) | 0.011 | 1.225(0.806,1.862) | 0.341 |
|  | ≥10 hours/d | | 3.625(2.047,6.420) | <0.001 | 2.959(1.716,5.100) | <0.001 |
| **Objective and Subjective**  **Sleep Duration**  **Interaction Group** | obj. ≥6 hours/d | subj. ≥6 hours/d | 1[Ref] |  | 1[Ref] |  |
|  | obj. <6 hours/d | subj. ≥6 hours/d | 1.200(0.540,2.665) | 0.655 | 0.972(0.456,2.074) | 0.942 |
|  | obj. ≥6 hours/d | subj. <6 hours/d | 1.394(0.928,2.094) | 0.109 | 1.058(0.672,1.667) | 0.807 |
|  | obj. <6 hours/d | subj. <6 hours/d | 1.867(0.498,6.990) | 0.354 | 1.617(0.444,5.889) | 0.466 |
| **Sleep Duration → PHQ-9 Score** | | | ***β* (95%CI)** | ***P* value** | ***β* (95%CI)** | ***P* value** |
| Sleep duration (obj.), hour/d | | | 0.177(0.089, 0.264) | <0.001 | 0.161(0.080, 0.241) | <0.001 |
| Sleep duration (subj.), hour/d | | | -0.397(-0.517, -0.276) | <0.001 | -0.343(-0.466, -0.221) | <0.001 |
| **PHQ-9 Score → Fatal MACE** | | | **HR (95%CI)** | ***P* value** | **HR (95%CI)** | ***P* value** |
| PHQ-9 score | | | 1.051(1.015,1.088) | 0.005 | 1.033(0.988,1.081) | 0.154 |

**Abbreviations:** BMI, body mass index; PHQ-9, Patient Health Questionnaire; MACE, major adverse cardiovascular events.

Adjusted for age, sex, race, education level, poverty/income ratio, current smoking, BMI, hypertension, and diabetes.

**Supplementary Table 5. Structural Equation Modeling: Effects of PHQ-9 Score on Sleep Duration (<7** **hours/day) and Fatal MACE.**

| **Objective Sleep Duration** | | | | | | | | | |
| --- | --- | --- | --- | --- | --- | --- | --- | --- | --- |
| **Sleep duration effect on PHQ-9 score (**a: X→M**)** | | | | | **PHQ-9 score effect on Fatal MACE (**b: M→Y**)** | | | | |
| *β* | Lower | Upper | *P* value | Standardized *β* | *β* | Lower | Upper | *P* value | Standardized *β* |
| -0.164 | -0.380 | 0.052 | 0.137 | -0.029 | 0.000 | -0.001 | 0.001 | 0.726 | 0.008 |
| **Indirect Effect (**a*b: X→M→Y**)** | | | | | **Direct Effect (**c': X→Y adj M**)** | | | | |
| *β* | Lower | Upper | *P* value | Standardized *β* | *β* | Lower | Upper | *P* value | Standardized *β* |
| -0.000 | 0.000 | 0.000 | 0.737 | -0.000 | -0.002 | -0.009 | 0.004 | 0.466 | -0.016 |
| **Mediated (%) (**a*b/c**)** | | | | | **Total Effect (**c: X→Y**)** | | | | |
| - | | | | | *β* | Lower | Upper | *P* value | Standardized *β* |
|  |  |  |  |  | -0.002 | -0.009 | 0.004 | 0.462 | -0.016 |
| **Subjective Sleep Duration** | | | | | | | | | |
| **Sleep duration effect on PHQ-9 score (**a: X→M**)** | | | | | **PHQ-9 score effect on Fatal MACE (**b: M→Y**)** | | | | |
| *β* | Lower | Upper | *P* value | Standardized *β* | *β* | Lower | Upper | *P* value | Standardized *β* |
| -1.127 | -1.366 | -0.888 | <0.001 | -0.189 | 0.001 | 0.000 | 0.002 | 0.038 | 0.045 |
| **Indirect Effect (**a*b: X→M→Y**)** | | | | | **Direct Effect (**c': X→Y adj M**)** | | | | |
| *β* | Lower | Upper | *P* value | Standardized *β* | *β* | Lower | Upper | *P* value | Standardized *β* |
| -0.001 | -0.003 | 0.000 | 0.044 | -0.009 | -0.001 | -0.007 | 0.005 | 0.704 | -0.008 |
| **Mediated (%) (**a*b/c**)** | | | | | **Total Effect (**c: X→Y**)** | | | | |
| 56.25% | | | | | *β* | Lower | Upper | *P* value | Standardized *β* |
|  |  |  |  |  | -0.003 | -0.009 | 0.004 | 0.443 | -0.016 |

**Abbreviations:** BMI, body mass index; PHQ-9, Patient Health Questionnaire; MACE, major adverse cardiovascular events.

Adjusted for age, sex, race, education level, poverty/income ratio, current smoking, BMI, hypertension, and diabetes.

**Supplementary Table 6. Structural Equation Modeling: Effects of PHQ-9 Score on Sleep Duration (**≥**7** **hours/day) and Fatal MACE.**

| **Objective Sleep Duration** | | | | | | | | | |
| --- | --- | --- | --- | --- | --- | --- | --- | --- | --- |
| **Sleep duration effect on PHQ-9 score (**a: X→M**)** | | | | | **PHQ-9 score effect on Fatal MACE (**b: M→Y**)** | | | | |
| *β* | Lower | Upper | *P* value | Standardized *β* | *β* | Lower | Upper | *P* value | Standardized *β* |
| 0.343 | 0.237 | 0.449 | <0.001 | 0.101 | 0.001 | -0.001 | 0.002 | 0.285 | 0.017 |
| **Indirect Effect (**a*b: X→M→Y**)** | | | | | **Direct Effect (**c': X→Y adj M**)** | | | | |
| *β* | Lower | Upper | *P* value | Standardized *β* | *β* | Lower | Upper | *P* value | Standardized *β* |
| 0.000 | 0.000 | 0.001 | 0.292 | 0.002 | 0.011 | 0.006 | 0.016 | <0.001 | 0.086 |
| **Mediated (%) (**a*b/c**)** | | | | | **Total Effect (**c: X→Y**)** | | | | |
| - | | | | | *β* | Lower | Upper | *P* value | Standardized *β* |
|  |  |  |  |  | 0.011 | 0.007 | 0.016 | <0.001 | 0.087 |
| **Subjective Sleep Duration** | | | | | | | | | |
| **Sleep duration effect on PHQ-9 score (**a: X→M**)** | | | | | **PHQ-9 score effect on Fatal MACE (**b: M→Y**)** | | | | |
| *β* | Lower | Upper | *P* value | Standardized *β* | *β* | Lower | Upper | *P* value | Standardized *β* |
| 0.429 | 0.259 | 0.598 | <0.001 | 0.098 | 0.000 | -0.001 | 0.002 | 0.596 | 0.008 |
| **Indirect Effect (**a*b: X→M→Y**)** | | | | | **Direct Effect (**c': X→Y adj M**)** | | | | |
| *β* | Lower | Upper | *P* value | Standardized *β* | *β* | Lower | Upper | *P* value | Standardized *β* |
| 0.000 | 0.000 | 0.001 | 0.595 | 0.001 | 0.016 | 0.008 | 0.023 | <0.001 | 0.085 |
| **Mediated (%) (**a*b/c**)** | | | | | **Total Effect (**c: X→Y**)** | | | | |
| - | | | | | *β* | Lower | Upper | *P* value | Standardized *β* |
|  |  |  |  |  | 0.016 | 0.008 | 0.023 | <0.001 | 0.085 |

**Abbreviations:** BMI, body mass index; PHQ-9, Patient Health Questionnaire; MACE, major adverse cardiovascular events.

Adjusted for age, sex, race, education level, poverty/income ratio, current smoking, BMI, hypertension, and diabetes.

**Supplementary Table 7. Structural Equation Modeling: Effects of PHQ-9 Score on Sleep Duration and All-Cause Mortality.**

| **Objective Sleep Duration** ≥**7** **hours/day** | | | | | | | | | | |
| --- | --- | --- | --- | --- | --- | --- | --- | --- | --- | --- |
| **Mediator** | **Sleep duration effect on Mediator (**a: X→M**)** | | | | | **Mediator effect on All-cause mortality (**b: M→Y**)** | | | | |
|  | *β* | Lower | Upper | *P* value | Standardized *β* | *β* | Lower | Upper | *P* value | Standardized *β* |
| PHQ-9 score | 0.343 | 0.237 | 0.449 | <0.001 | 0.101 | 0.002 | 0.001 | 0.004 | 0.042 | 0.029 |
| Physical activity, h/w | -0.916 | -1.330 | -0.501 | <0.001 | -0.057 | -0.001 | -0.001 | 0.000 | <0.001 | -0.040 |
| Coffee, cup | -0.081 | -0.130 | -0.031 | 0.002 | -0.044 | -0.002 | -0.006 | 0.001 | 0.202 | -0.020 |
| Alcohol, cup | -0.017 | -0.057 | 0.023 | 0.400 | -0.011 | -0.002 | -0.005 | 0.001 | 0.234 | -0.014 |
| **Mediator** | **Indirect Effect (**a*b: X→M→Y**)** | | | | | **Direct Effect (**c': X→Y adj M**)** | | | | |
|  | *β* | Lower | Upper | *P* value | Standardized *β* | *β* | Lower | Upper | *P* value | Standardized *β* |
| PHQ-9 score | 0.001 | 0.000 | 0.001 | 0.022 | 0.003 | 0.030 | 0.023 | 0.037 | <0.001 | 0.136 |
| Physical activity, h/w | 0.000 | 0.000 | 0.001 | 0.001 | 0.002 | **Total Effect (**c: X→Y**)** | | | | |
| Coffee, cup | 0.000 | 0.000 | 0.001 | 0.234 | 0.001 | *β* | Lower | Upper | *P* value | Standardized *β* |
| Alcohol, cup | 0.000 | 0.000 | 0.000 | 0.458 | 0.000 | 0.031 | 0.024 | 0.038 | <0.001 | 0.143 |
| **Subjective Sleep Duration** <**7** **hours/day** | | | | | | | | | | |
| **Mediator** | **Sleep duration effect on PHQ-9 score (**a: X→M**)** | | | | | **PHQ-9 score effect on All-cause mortality (**b: M→Y**)** | | | | |
|  | *β* | Lower | Upper | *P* value | Standardized *β* | *β* | Lower | Upper | *P* value | Standardized *β* |
| PHQ-9 score | -1.127 | -1.366 | -0.888 | <0.001 | -0.189 | 0.003 | 0.001 | 0.005 | 0.003 | 0.066 |
| Physical activity, h/w | -2.593 | -3.861 | -1.325 | <0.001 | -0.084 | -0.000 | -0.001 | 0.000 | 0.001 | -0.045 |
| Coffee, cup | 0.005 | -0.109 | 0.118 | 0.937 | 0.002 | -0.000 | -0.004 | 0.004 | 0.981 | 0.000 |
| Alcohol, cup | -0.056 | -0.151 | 0.038 | 0.243 | -0.022 | -0.000 | -0.004 | 0.004 | 0.924 | -0.002 |
| **Mediator** | **Indirect Effect (**a*b: X→M→Y**)** | | | | | **Direct Effect (**c': X→Y adj M**)** | | | | |
|  | *β* | Lower | Upper | *P* value | Standardized *β* | *β* | Lower | Upper | *P* value | Standardized *β* |
| PHQ-9 score | -0.004 | -0.006 | -0.001 | 0.004 | -0.012 | -0.007 | -0.019 | 0.005 | 0.247 | -0.023 |
| Physical activity, h/w | 0.001 | 0.000 | 0.002 | 0.012 | 0.004 | **Total Effect (**c: X→Y**)** | | | | |
| Coffee, cup | 0.000 | 0.000 | 0.000 | 0.981 | 0.000 | *β* | Lower | Upper | *P* value | Standardized *β* |
| Alcohol, cup | 0.000 | 0.000 | 0.000 | 0.924 | 0.000 | -0.009 | -0.021 | 0.000 | 0.050 | -0.032 |

**Abbreviations:** BMI, body mass index; PHQ-9, Patient Health Questionnaire.

Adjusted for age, sex, race, education level, poverty/income ratio, current smoking, BMI, hypertension, and diabetes.
